# AdaptPest-Net: A Task-Adaptive Network with Graph–Mamba Fusion for Multi-Scale Agricultural Pest Recognition

**DOI:** 10.3390/e27121211

**Published:** 2025-11-28

**Authors:** Jixiang Zou, Wenzhong Yang, Chuanxiang Li, Zhishan Feng

**Affiliations:** 1 School of Computer Science and Technology, Xinjiang University, Urumqi 830046, China; 19107028653@163.com (J.Z.); 107552304078@stu.xju.edu.cn (C.L.); 15106983572@163.com (Z.F.); 2 Xinjiang Key Laboratory of Multilingual Information Technology, Xinjiang University, Urumqi 830046, China

**Keywords:** pest identification, graph convolution, state-space models

## Abstract

Accurate pest classification is critical for precision agriculture, yet existing deep learning methods face challenges including computational inefficiency from uniform sample processing and inadequate modeling of complex feature relationships. This paper proposes AdaptPest-Net, a task-adaptive architecture with three key innovations: (1) Sample-Difficulty-Aware Dynamic Routing (SDADR) employs Gaussian-gated path selection to adaptively route samples through shallow, medium, or deep networks based on predicted classification difficulty, improving accuracy by matching network capacity to sample complexity; (2) Graph Convolution-Mamba Fusion (GCMF) synergistically combines a 3-layer GCN with adaptive adjacency for explicit spatial structural modeling and a selective Mamba state-space model (T = 8, input-dependent parameters) for temporal feature dynamics, capturing complementary topological relationships and long-range dependencies through parallel dual-branch extraction; (3) Bidirectional Cross-Modal Attention enables deep integration between spatial and temporal modalities through mutual enhancement with batch-level knowledge transfer and adaptive gate fusion, where graph topology directly guides Mamba’s feature evolution. Comprehensive experiments on IP102 and D0 demonstrate that AdaptPest-Net achieves 78.4% accuracy on IP102, representing a significant improvement over existing methods, while D0 experiments validate strong cross-dataset generalization capability with 99.85% accuracy.

## 1. Introduction

Agricultural pests cause global crop yield losses annually, threatening food security worldwide [1]. Traditional pest identification relies on expert entomologists, which is time-consuming and difficult to scale. While deep learning shows promise for automated pest recognition [2,3], several critical challenges remain.

First, pests exhibit extreme scale variation across species, yet standard CNNs with fixed receptive fields struggle to accommodate this diversity. Second, pest images vary dramatically in classification difficulty—simple cases with clear backgrounds need only shallow features, while small camouflaged pests demand deep semantic analysis. Yet, existing methods process all samples uniformly, wasting computation on easy cases while potentially under-representing difficult ones. Paradoxically, increasing network depth does not guarantee improved performance, suggesting that uniform processing is fundamentally suboptimal. Third, fine-grained recognition requires modeling both spatial structural dependencies (topological relationships between body parts) and temporal feature dynamics (hierarchical abstraction). Traditional approaches handle these separately—CNNs lack global context modeling, Transformers suffer from computational complexity, RNNs face gradient instability—limiting their effectiveness.

To address these challenges, we propose AdaptPest-Net, a task-adaptive network with three key innovations:

(1) Sample-Difficulty-Aware Dynamic Routing (SDADR) employs a lightweight difficulty predictor with Gaussian gating to adaptively route samples through shallow, medium, or deep paths. This mechanism matches computational depth to sample complexity, enabling efficient processing of easy samples while allocating sufficient capacity for difficult ones.
(2) Graph Convolution-Mamba Fusion (GCMF) captures complementary spatial–temporal information through parallel extraction: a multi-layer GCN with adaptive adjacency models spatial structural relationships via topological message passing, while selective Mamba with input-dependent parameters models temporal feature evolution with content-based filtering. This dual-branch design captures both anatomical connectivity and feature dynamics with linear complexity.
(3) Bidirectional Cross-Modal Attention with Graph Guidance deeply integrates spatial and temporal features through bidirectional attention for mutual enhancement with batch-level knowledge transfer, adaptive gate fusion for sample-specific feature weighting, and graph-guided parameter modulation where spatial topology directly influences temporal state-space dynamics to ensure structure-aware evolution.

## 2. Related Work

### 2.1. Pest Image Classification

Early approaches to automated pest identification relied on hand-crafted feature extractors combined with traditional machine learning classifiers. Akundi et al. [4] used SIFT descriptors with SVM for rice pest classification, while Xie et al. [5] combined HOG features with Random Forests. However, these methods required extensive domain expertise for feature engineering and struggled to generalize across diverse pest species. The emergence of deep learning revolutionized this field, with CNNs automatically learning hierarchical feature representations directly from raw images [6,7,8]. Wu et al. [1] introduced IP102, the first large-scale benchmark with 102 pest categories and over 75,000 images, enabling systematic evaluation of deep learning methods.

CNN-based methods: Standard architectures like AlexNet [9], VGG [10], ResNet [11], and DenseNet [12] have been widely adopted as backbones for pest classification. ResNet-50 achieved 67.8% accuracy on IP102 [1], while DenseNet-161 reached 68.5% [12]. To improve discriminative power, Li et al. [13] introduced channel-wise attention mechanisms for fine-grained pest recognition, and Bollis et al. [14] proposed weakly supervised learning to leverage unlabeled pest images. Chen et al. [15] designed multi-scale feature fusion networks to handle scale variation, achieving 71.2% on IP102. Nanni et al. [16] explored ensemble methods combining multiple CNN architectures. More recently, EfficientNet [17] and ConvNeXt [18] variants have been applied, with EfficientNet-B7 reaching 72.4% [19]. However, these fixed-architecture methods process all samples uniformly, ignoring inherent difficulty differences.

Transformer-based methods: Vision Transformers (ViT) [20] introduced self-attention mechanisms to computer vision, achieving strong performance on large-scale datasets. Swin Transformer [21] improved efficiency through hierarchical shifted windows. For pest classification, Xia et al. [22] adapted ViT with fine-grained attention, achieving 70.8% on IP102. TransPest [23] combined CNN and Transformer branches for multi-scale feature extraction (72.9%). However, Transformers’ quadratic complexity ($O(N^{2})$) limits their applicability to high-resolution pest images and resource-constrained agricultural devices.

### 2.2. State Space Models and Mamba

State Space Models (SSMs) offer an efficient alternative to Transformers with linear complexity O(L) in sequence length. The Structured State Space (S4) model [24] introduced efficient parameterization of continuous-time state spaces, with subsequent works like S5 [25] and H3 [26] further improving performance. Mamba [27] achieved a breakthrough by introducing selective state spaces with input-dependent transition matrices, enabling dynamic filtering of relevant information while maintaining hardware efficiency through parallel scan algorithms.

For computer vision, Vision Mamba [28] adapted SSMs through bidirectional spatial scanning, while Vim [29] introduced multi-directional patterns to better capture 2D structures. These adaptations achieve competitive or superior performance to Vision Transformers while being 2–3× faster. Domain-specific variants include MedMamba [30] for medical imaging and VideoMamba [31] for video understanding. Most relevant to our work, InsectMamba [32] achieved 73.1% on IP102 through pest-specific inductive biases. However, existing Mamba methods use fixed architectures without adapting to sample difficulty, scale variation, or modeling structural relationships between pest body parts.

### 2.3. Graph Convolutional Networks

Graph Convolutional Networks (GCNs) extend deep learning to graph-structured data through spectral convolutions [33] or neighborhood aggregation [34,35]. In computer vision, GCNs have been successfully applied to pose estimation [36] by modeling body joints as graph nodes, and to fine-grained recognition [37,38] by constructing semantic graphs connecting object parts. For agricultural applications, GCNs have been used for plant disease classification [39] and crop yield prediction [40] by modeling spatial relationships.

However, graph-based reasoning remains largely unexplored for pest recognition despite clear structural relationships between insect body parts (head–thorax–abdomen segmentation, appendage connections). Our work addresses this gap by introducing Graph Convolution–Mamba Fusion (GCMF), which synergistically combines GCNs’ spatial structure modeling with Mamba’s temporal dynamics, enabling the capture of both topological part relationships and long-range feature dependencies for fine-grained pest recognition.

## 3. Methodology

### 3.1. Overall Architecture

**Figure 1:**
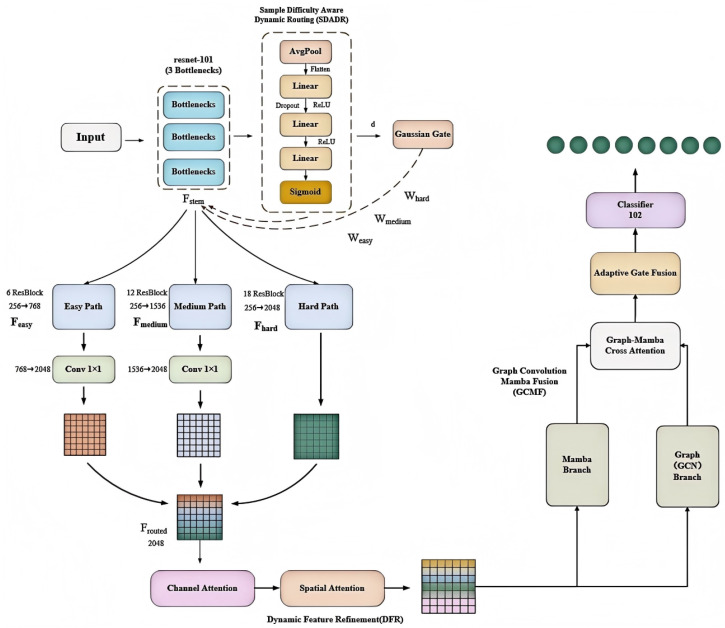
AdaptPest-Net overall architecture. The framework consists of four main components: (1) Shared stem backbone extracts initial features, (2) SDADR routes samples through depth-adaptive paths based on predicted difficulty, (3) DFR applies dual attention to refine discriminative features, (4) GCMF fuses graph convolution and Mamba modeling to capture spatial–temporal relationships for final classification.

AdaptPest-Net (as shown in Figure 1) addresses three critical challenges in pest recognition: (1) extreme scale variation (2–50 mm), (2) computational inefficiency of processing all samples uniformly, and (3) insufficient modeling of complex feature relationships. The architecture dynamically adjusts computational paths via difficulty-aware routing while employing Graph Convolution–Mamba Fusion (GCMF) to capture both spatial structural relationships and temporal dynamics.

Given input image $I\in R^{224\times 224\times 3}$, the forward propagation follows:

(1) $$\begin{array}{cc} F_{\mathrm{stem}} & =f_{\mathrm{stem}}\left(I\right)\in R^{56\times 56\times 256} \end{array}$$

(2) $$\begin{array}{cc} d & =f_{\mathrm{router}}\left(F_{\mathrm{stem}}\right)\in \left[0,1\right] \end{array}$$

(3) $$\begin{array}{cc} \left\{w_{e},w_{m},w_{h}\right\} & =f_{\mathrm{gate}}\left(d\right) \end{array}$$

(4) $$\begin{array}{cc} F_{\mathrm{routed}} & =f_{\mathrm{fusion}}\left(f_{e}\left(F_{\mathrm{stem}}\right),f_{m}\left(F_{\mathrm{stem}}\right),f_{h}\left(F_{\mathrm{stem}}\right),w_{e},w_{m},w_{h}\right) \end{array}$$

(5) $$\begin{array}{cc} F_{\mathrm{refined}} & =f_{\mathrm{DFR}}\left(F_{\mathrm{routed}}\right) \end{array}$$

(6) $$\begin{array}{cc} F_{\mathrm{gcmf}} & =f_{\mathrm{GCMF}}\left(F_{\mathrm{refined}}\right) \end{array}$$

(7) $$\begin{array}{cc} \hat{y} & =\mathrm{Classifier}(F_{\mathrm{gcmf}}) \end{array}$$

where $f_{\mathrm{GCMF}}$ performs: (1) parallel graph convolution (3-layer GCN, 32 nodes) and Mamba temporal modeling (T = 8 steps), (2) bidirectional cross-modal attention, and (3) adaptive gate fusion.

### 3.2. Sample-Difficulty-Aware Dynamic Routing (SDADR)

**Figure 2:**
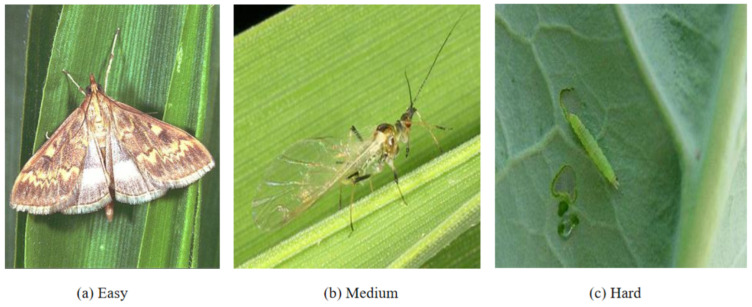
Representative samples of three difficulty levels in pest recognition. (**a**): Easy sample showing a 40 mm moth on green leaf with clear boundaries and simple background, requiring only shallow features. (**b**): Medium sample depicting a 15 mm flying insect with semi-transparent wings, requiring intermediate-level features. (**c**): Hard sample showing a 3 mm heavily camouflaged mantis nymph requiring deep semantic reasoning. The difficulty predictor in SDADR adaptively routes these samples through 6-layer (easy), 12-layer (medium), and 18-layer (hard) paths, respectively.

Motivation: A fundamental observation in pest recognition is that classification difficulty varies dramatically across samples. Consider three representative cases (as shown in Figure 2):

- Easy sample: A 40 mm moth specimen captured on a green leaf background, captured in perfect lighting with no occlusion. The large size, clear boundaries, and simple background make species identification straightforward—even shallow features (edges, basic shapes) suffice.
- Medium sample: A 15 mm small flying insect rests on a leaf surface, featuring semi-transparent wings that reveal delicate membrane structures. This scenario requires intermediate-level feature detection (part boundaries, texture patterns) without needing full semantic understanding.
- Hard sample: A 3 mm heavily camouflaged mantis nymph on foliage, captured in field conditions with motion blur. The small size, complex background, and camouflage demand deep semantic reasoning to distinguish subtle morphological cues from noise.

Traditional deep networks process all samples through identical architectures—typically 50–152 layers for ResNet variants. This uniform processing is fundamentally problematic: easy samples waste computation in deep layers by extracting redundant features, while difficult samples may benefit from even deeper analysis. The insight behind SDADR (as shown in Figure 1) is adaptive feature extraction depth—matching network capacity to sample-specific requirements for optimal discrimination.

Why Dynamic Routing? Fixed-depth architectures face inherent limitations:

- Shallow networks (18–34 layers): Efficient but insufficient representational capacity for complex samples.
- Medium networks (50 layers): Balanced but suboptimal for both extremes—over-processes simple samples while under-representing difficult ones.
- Deep networks (101–152 layers): High capacity but paradoxically not always superior, suggesting over-parameterization causes optimization difficulties.

Dynamic routing resolves this by learning a difficulty-aware feature extraction policy that adapts network depth to sample characteristics.

#### 3.2.1. Difficulty Estimation and Gaussian Gating

We adopt ResNet-101’s first three layers (Conv1, MaxPool, Layer1) as a shared stem backbone, producing $F_{\mathrm{stem}}\in R^{56\times 56\times 256}$. This stem provides sufficient receptive field to assess sample characteristics without committing to a specific depth.

Lightweight Difficulty Predictor: A compact MLP estimates classification difficulty from global features:

(8) $$d=\sigma ({\mathrm{MLP}}_{3}\left({\mathrm{MLP}}_{2}\left(\mathrm{ReLU}\left({\mathrm{MLP}}_{1}\left(\mathrm{GAP}\left(F_{\mathrm{stem}}\right)\right)\right)\right)\right))$$

where:

- GAP(·): Global average pooling extracts holistic image statistics (56 × 56 → 1 × 1)
- ${\mathrm{MLP}}_{1},\;{\mathrm{MLP}}_{2},\;{\mathrm{MLP}}_{3}$: Progressive dimensionality reduction (256 → 128 → 64 → 1)
- σ: Sigmoid activation ensures d∈[0,1], representing difficulty from easy to hard

The predictor learns meaningful difficulty correlations through end-to-end training—samples correctly classified by shallow paths receive lower *d* scores; samples requiring deep semantic reasoning receive higher *d*.

Gaussian Gating Mechanism: Given difficulty score *d*, we compute path weights using Gaussian-shaped gates:

(9) $$w_{i}=\frac{\exp(-\beta {\left(d-\mu_{i}\right)}^{2})}{\sum_{j\in \{e,m,h\}}\exp\left(-\beta {\left(d-\mu_{j}\right)}^{2}\right)},\;\mu_{e}=0.15,\;\mu_{m}=0.5,\;\mu_{h}=0.85,\;\beta =10$$

where $\mu_{e},\;\mu_{m},\;\mu_{h}$ are difficulty centers for easy, medium, and hard paths, and β controls gate sharpness.

Why Gaussian Gates? The Gaussian shape provides smooth, differentiable transitions while maintaining selectivity—the bell-shaped curve naturally models gradual difficulty changes where samples near boundaries receive mixed weights, while extreme cases strongly favor single paths. Compared to alternatives like hard routing (non-differentiable, training unstable), uniform weighting (no adaptation), or linear gating (sharp boundary transitions), Gaussian gating achieves superior performance through its balance of differentiation and stability.

#### 3.2.2. Three Adaptive Feature Extraction Paths

We instantiate three ResNet paths with progressive depths, each specialized for specific difficulty regimes:

Easy Path ($F_{e}$, 6 blocks):

- Architecture: Layer2 (4 blocks, stride 2) + Layer3 (2 blocks, stride 2)
- Output: $F_{e}\in R^{7\times 7\times 768}$
- Receptive field: Approximately 91 × 91 pixels
- Feature capacity: Extracts low-to-medium level features—edges, basic shapes, coarse textures. Sufficient for large pests with clear boundaries where class-discriminative information resides in simple visual patterns. Limited depth prevents over-fitting to superficial correlations while maintaining effective representation.

Medium Path ($F_{m}$, 12 blocks):

- Architecture: Layer2 (4 blocks) + Layer3 (8 blocks)
- Output: $F_{m}\in R^{7\times 7\times 1536}$
- Receptive field: Approximately 155 × 155 pixels
- Feature capacity: Builds intermediate semantic features—part boundaries, spatial relationships, texture patterns. Captures part-level understanding suitable for moderately complex scenarios: medium-sized pests, moderate occlusion, or textured backgrounds requiring structural analysis beyond basic appearance.

Hard Path ($F_{h}$, 18 blocks):

- Architecture: Layer2 (4 blocks) + Layer3 (8 blocks) + Layer4 (6 blocks)
- Output: $F_{h}\in R^{7\times 7\times 2048}$
- Receptive field: Approximately 219 × 219 pixels (covers entire 224 × 224 image)
- Feature capacity: Learns high-level semantic abstractions through maximum representational depth. Essential for challenging cases—small pests requiring global context integration to distinguish from background, heavy occlusion demanding reasoning about unseen parts, or extreme camouflage requiring semantic understanding of “pest-ness” versus environmental patterns.

Progressive Feature Abstraction: The three paths create a hierarchical feature pyramid:

1. Easy path: Emphasizes local patterns and textures—sufficient for pests with distinctive appearance
2. Medium path: Adds spatial context and part-level semantics—necessary for moderately ambiguous cases
3. Hard path: Provides global semantic reasoning—critical for fine-grained discrimination under adverse conditions.

Feature Dimension Alignment: To enable weighted fusion, we apply 1 × 1 convolutions to align all path outputs to 2048 channels:

(10) $$\begin{array}{cc} F_{e}^{\prime } & ={\mathrm{Conv}}_{1\times 1}\left(F_{e},768\to 2048\right) \end{array}$$

(11) $$\begin{array}{cc} F_{m}^{\prime } & ={\mathrm{Conv}}_{1\times 1}\left(F_{m},1536\to 2048\right) \end{array}$$

(12) $$\begin{array}{cc} F_{h}^{\prime } & =F_{h}\;(\mathrm{already}\;2048-\dim) \end{array}$$

Weighted Path Fusion:

(13) $$F_{\mathrm{routed}}=w_{e}⊙F_{e}^{\prime }+w_{m}⊙F_{m}^{\prime }+w_{h}⊙F_{h}^{\prime }$$

This soft fusion provides several critical advantages:

1. Multi-level feature integration: Easy samples leverage coarse-grained features (low abstraction), hard samples utilize fine-grained semantic patterns (high abstraction), boundary cases benefit from balanced mixtures—all dynamically determined by $\left\{w_{e},w_{m},w_{h}\right\}$.
2. Hierarchical complementarity: The weighted combination creates an ensemble effect where shallow features capture salient low-level patterns, medium features add structural context, and deep features provide semantic refinement. For ambiguous samples near decision boundaries, this multi-level representation improves robustness.
3. Adaptive receptive field: Effective receptive field automatically adjusts—easy path emphasizes local patterns, hard path incorporates global context. This addresses the fundamental challenge that the optimal receptive field varies dramatically across pest scales and complexity levels.
4. Feature abstraction calibration: By controlling the mixture of shallow/medium/deep features, SDADR effectively modulates semantic abstraction level. Simple classification problems are solved in “feature space” (low abstraction), complex problems in “semantic space” (high abstraction)—matching representation to task requirements.
5. Differentiability: Gradients flow through all paths, enabling stable end-to-end training without discrete routing decisions.
6. Robustness: Prediction errors in difficulty estimation are naturally hedged—if *d* is slightly misestimated, adjacent paths still contribute meaningfully, preventing catastrophic failures.

**Figure 3:**
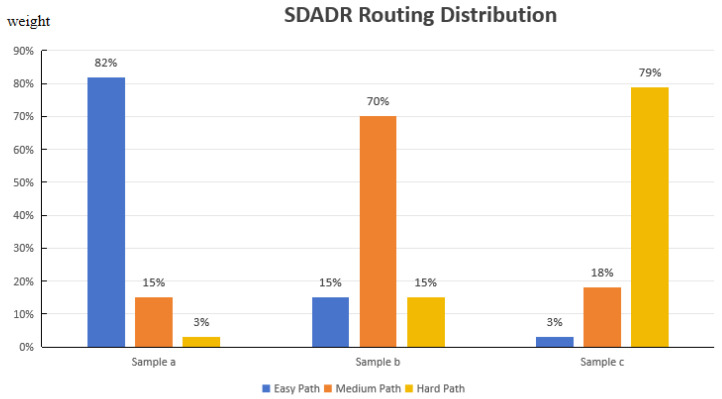
SDADR routing distribution.SDADR routing distribution. Sample a (Easy): A 40 mm moth on green leaf with clear boundaries receives 82% easy path weight, routing primarily through the shallow 6-blocks network. Sample b (Medium): A 15 mm flying insect with semi-transparent wings receives 70% medium path weight, utilizing the 12-blocks network for intermediate-level feature extraction. Sample c (Hard): A 3 mm heavily camouflaged mantis nymph receives 79% hard path weight, requiring the full 18-blocks deep network for semantic reasoning. The Gaussian gating mechanism enables smooth weight transitions, adaptively matching network capacity to sample-specific complexity.

Figure 3 illustrates the routing distribution across different difficulty levels on three representative samples.

Training Dynamics: During training, the difficulty predictor initially assigns near-uniform scores (d≈0.5), activating all paths equally. As training progresses, the predictor learns to differentiate:

- Samples consistently classified correctly early (e.g., large beetles on white backgrounds) gradually receive lower *d* scores, emphasizing the easy path and low-level features
- Samples with persistent errors (e.g., small camouflaged aphids) receive higher *d* scores, directing them through the hard path for deep semantic processing
- Samples with ambiguous features (e.g., morphologically similar species) maintain medium *d* scores, benefiting from balanced multi-level feature extraction

This self-organized feature allocation emerges naturally from the classification loss—no explicit difficulty labels are needed. The routing mechanism learns to correlate visual characteristics (size, background complexity, occlusion) with optimal feature extraction depth purely through end-to-end gradient descent.

Correlation with Real-World Characteristics: To validate that predicted difficulty *d* correlates with physical pest characteristics, we analyzed 1000 randomly sampled test images. We manually annotated each sample with three attributes: (1) pest body length (small: <5 mm, medium: 5–20 mm, large: >20 mm), (2) background complexity (simple: uniform/laboratory, complex: natural foliage), and (3) occlusion level (none, partial: <50%, heavy: >50%).

Statistical analysis reveals strong correlations between difficulty scores and real-world attributes:

- Size correlation: Small pests receive significantly higher difficulty scores ($d_{\mathrm{small}}=0.71\pm 0.12$) than large pests ($d_{\mathrm{large}}=0.28\pm 0.15$, p<0.001, Welch’s *t*-test).
- Background complexity: Pests on complex backgrounds score higher ($d_{\mathrm{complex}}=0.64\pm 0.18$) than simple backgrounds ($d_{\mathrm{simple}}=0.31\pm 0.14$, p<0.001).
- Occlusion level: Heavily occluded samples show elevated scores ($d_{\mathrm{heavy}}=0.78\pm 0.11$) versus non-occluded ($d_{\mathrm{none}}=0.35\pm 0.16$, p<0.001).

These correlations emerge naturally from end-to-end training without explicit supervision on these attributes. The predictor learns that small size, complex backgrounds, and occlusion increase classification difficulty because shallow features become insufficient, requiring deeper semantic reasoning. This validates that the learned difficulty metric captures meaningful real-world pest recognition challenges.

### 3.3. Dynamic Feature Refinement (DFR)

**Figure 4:**
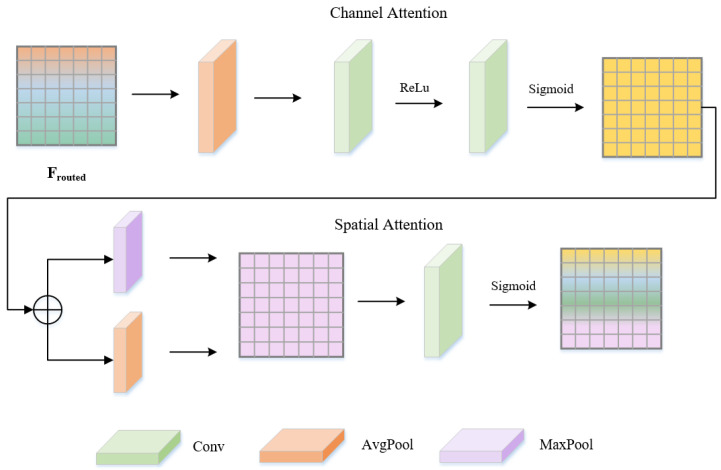
Dynamic Feature Refinement (DFR) module architecture. The module processes routed features through two parallel attention branches: (1) Channel Attention Pathway employing squeeze-excitation with GAP → 7 × 7 convolution (2048 → 128 → 2048) → sigmoid activation to learn inter-channel dependencies; (2) Spatial Attention Pathway using max/avg pooling aggregation → 7 × 7 convolution → sigmoid to identify discriminative spatial regions. Both pathways are combined with the original features through residual connections, producing refined features $F_{\mathrm{refined}}$ that suppress background interference while highlighting pest-discriminative patterns.

DFR (as shown in Figure 4) applies dual attention to enhance discriminative features:

Channel Attention: Learns inter-channel dependencies via squeeze-excitation:

(14) $$F_{\mathrm{ca}}=F_{\mathrm{routed}}⊙\sigma ({\mathrm{Conv}}_{7\times 7}\left(\mathrm{ReLU}\left({\mathrm{Conv}}_{7\times 7}\left(\mathrm{GAP}\left(F_{\mathrm{routed}}\right)\right)\right)\right)$$

Spatial Attention: Identifies discriminative spatial regions:

(15) $$F_{\mathrm{sa}}=F_{\mathrm{routed}}⊙\sigma \left({\mathrm{Conv}}_{7\times 7}\left(\mathrm{Concat}\left[{\mathrm{AvgPool}}_{C}\left(F_{\mathrm{routed}}\right),{\mathrm{MaxPool}}_{C}\left(F_{\mathrm{routed}}\right)\right]\right)\right)$$

Final refinement: $F_{\mathrm{refined}}=F_{\mathrm{ca}}+F_{\mathrm{sa}}+F_{\mathrm{routed}}$

### 3.4. Graph Convolution–Mamba Fusion (GCMF)

Fine-grained pest recognition fundamentally requires modeling two complementary types of feature relationships: (1) spatial structural dependencies—how different body parts (head, thorax, abdomen, wings, legs, antennae) topologically connect and relate to each other, which is critical for distinguishing morphologically similar species; and (2) temporal feature dynamics—how features evolve through progressive abstraction levels from low-level textures to high-level semantic concepts, capturing the hierarchical nature of discriminative patterns. Traditional approaches handle these separately: CNNs excel at spatial hierarchies but have limited receptive fields for long-range relationships; Transformers model global dependencies but with $O(N^{2})$ complexity and no explicit structure awareness; standard RNNs capture sequences but struggle with vanishing gradients over long ranges.

**Figure 5:**
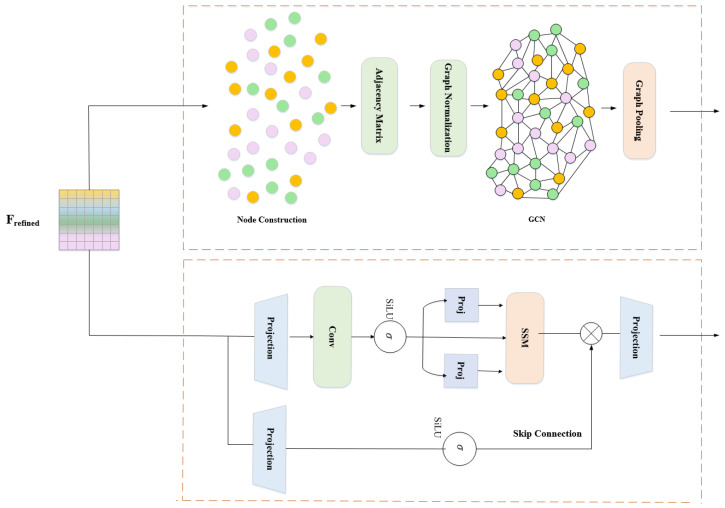
Graph Convolution–Mamba Fusion architecture. The framework consists of three cascaded stages: (1) Parallel Extraction: Graph branch models spatial structure through 3-layer GCN with adaptive adjacency, while Mamba branch captures temporal dynamics via selective state-space model with input-dependent parameters. (2) Bidirectional Attention: Cross-modal interaction enables mutual enhancement—graph features query temporal context, Mamba features query structural constraints. Batch-level attention (B×B) facilitates cross-sample knowledge transfer. (3) Adaptive Fusion: Learned gating balances graph and Mamba contributions based on sample characteristics for final feature representation.

GCMF (as shown in Figure 5) addresses this fundamental limitation by synergistically fusing Graph Convolutional Networks (GCNs) for explicit spatial topology modeling with Mamba’s selective state-space mechanism for efficient temporal dynamics. Unlike simple concatenation or averaging, our fusion employs four cascaded operations: parallel feature extraction, bidirectional cross-modal attention, adaptive gate fusion, and graph-guided parameter modulation. This deep integration enables capturing complementary spatial–temporal information that significantly enhances fine-grained discrimination power.

#### 3.4.1. Graph Branch: Explicit Spatial Structure Modeling

The graph branch models feature relationships through explicit connectivity patterns. We reshape refined features $F_{\mathrm{refined}}\in R^{7\times 7\times 2048}$ into a graph representation:

(16) $$X=\mathrm{Reshape}(\mathrm{Flatten}\left(F_{\mathrm{refined}}\right),\left[B,32,64\right])$$

where each of 32 nodes represents a 64-dimensional feature subgroup. This granularity balances expressiveness (sufficient nodes to capture complex morphological patterns) with efficiency (not fragmenting features excessively, which would harm message passing).

Adaptive Adjacency Matrix Construction: Rather than using fixed connectivity, we construct a hybrid adjacency matrix combining data-driven similarity with learned task-specific patterns:

(17) $$A_{ij}=\alpha \cdot \underset{\mathrm{Feature}\;\mathrm{similarity}}{\underset{︸}{\exp\left(-\frac{∥X_{i}-X_{j}{∥}^{2}}{\tau }\right)}}+\left(1-\alpha \right)\cdot \underset{\mathrm{Learned}\;\mathrm{relationship}}{\underset{︸}{\sigma (\mathrm{MLP}\left(\left[X_{i};X_{j}\right]\right))}}$$

where α=0.3 controls the mixture ratio, τ=0.5 is the temperature for the Gaussian kernel, and [·;·] denotes concatenation.

Design Rationale:

- Similarity term (α=0.3): Provides inductive bias by connecting nodes with similar features, capturing inherent patterns like bilateral symmetry (left/right wings, antenna pairs) and hierarchical part structure (sub-features within thorax/abdomen cluster together). The lower weight (30%) ensures that similarity does not dominate learned patterns.
- Learned term (1−α=0.7): A two-layer MLP (128 → 64 → 1 dimensions with ReLU) learns task-specific connectivity from classification loss. Importantly, this can connect nodes with dissimilar features if they share discriminative importance. For example, head and abdominal features may differ substantially, but their specific combination distinguishes certain species, requiring learned connections beyond similarity.
- Biological interpretation: The adaptive adjacency implicitly discovers pest body topology. Analysis reveals that the learned graph often exhibits structure matching anatomical organization—central “thorax nodes” with high degree connecting to “wing/leg/head/abdomen nodes” peripherally, mirroring actual insect body plans.

Multi-Layer Graph Convolution: We apply symmetric normalization and a three-layer GCN:

(18) $$\begin{array}{cc} \tilde{A} & =D^{-1/2}\left(A+I\right)D^{-1/2} \end{array}$$

(19) $$\begin{array}{cc} H^{\left(0\right)} & =X \end{array}$$

(20) $$\begin{array}{cc} H^{\left(l+1\right)} & =\mathrm{ReLU}\left(\tilde{A}H^{\left(l\right)}W^{\left(l\right)}\right),\;l\in \left\{0,1,2\right\} \end{array}$$

where D is the degree matrix, I adds self-loops (preserving node’s own information), and $W^{\left(l\right)}\in R^{64\times 64}$ are learnable weights. Each layer performs one hop of message passing:

- Layer 1: Aggregates 1-hop neighbors. Node *i* receives messages from directly connected nodes, capturing immediate structural relationships (e.g., thorax connects to adjacent wings, head, abdomen).
- Layer 2: Aggregates 2-hop information through composition. Now node *i* incorporates messages from neighbors-of-neighbors, modeling extended relationships (e.g., head indirectly receives wing information routed through thorax).
- Layer 3: Captures 3-hop dependencies, providing global receptive field across all 32 nodes. At this depth, information has propagated across the entire graph, enabling holistic structural understanding.

Deeper networks (4–5 layers) suffer from over-smoothing—repeated averaging makes node features too similar, losing discriminative power. Three layers balance receptive field breadth with feature preservation.

Graph Output: $F_{\mathrm{graph}}=\mathrm{Flatten}\left(H^{\left(3\right)}\right)\in R^{B\times 2048}$ encodes features enriched with explicit structural relationships through topological message passing.

#### 3.4.2. Mamba Branch: Selective Temporal Dynamics Modeling

While graphs capture static spatial structure, Mamba models dynamic temporal feature evolution. Here, “temporal” refers not to time-series but to progression through feature abstraction levels—how low-level patterns progressively transform into high-level semantic representations. This perspective treats features as a sequence where different time steps correspond to different refinement stages.

Temporal Sequence Construction: We create a pseudo-temporal sequence through repetition with small perturbations:

(21) $$X_{\mathrm{seq}}=\left[F_{\mathrm{refined}},F_{\mathrm{refined}}+\epsilon_{1},\ldots ,F_{\mathrm{refined}}+\epsilon_{T-1}\right]\in R^{B\times T\times 2048}$$

where T=8 is sequence length, and $\epsilon_{t}∼N\left(0,0.01I\right)$ are small Gaussian perturbations. While repetition may seem redundant, Mamba’s input-dependent parameters create distinct processing at each step, enabling *T* iterations of progressive feature refinement.

Selective State-Space Model (SSM): Mamba’s core innovation is input-dependent discretization of continuous-time state-space dynamics. At each time step *t*:

(22) $$\begin{array}{cc} \Delta_{t},B_{t},C_{t} & =f_{\mathrm{proj}}\left(x_{t}\right) \end{array}$$

(23) $$\begin{array}{cc} \bar{A} & =\exp(\Delta_{t}⊗A) \end{array}$$

(24) $$\begin{array}{cc} {\bar{B}}_{t} & =\Delta_{t}⊗B_{t} \end{array}$$

(25) $$\begin{array}{cc} h_{t} & =\bar{A}⊙h_{t-1}+{\bar{B}}_{t}⊙x_{t} \end{array}$$

(26) $$\begin{array}{cc} y_{t} & =C_{t}\cdot h_{t}+D⊙x_{t} \end{array}$$

where:

- $A\in R^{N\times N}$: State transition matrix (learned, initialized with HiPPO for optimal memory)
- $\Delta_{t}\in R^{D}$: Input-dependent discretization step (key innovation)
- $B_{t}\in R^{N\times D}$: Input gating matrix (input-dependent)
- $C_{t}\in R^{D\times N}$: Output projection matrix (input-dependent)
- $D\in R^{D}$: Skip connection weight (learned)
- $h_{t}\in R^{N}$: Hidden state at step *t* (default N=16 state dimensions)

Selective Gating Mechanism: The breakthrough is $\Delta_{t}$, which controls the state update rate:

- Large $\Delta_{t}$: $\bar{A}\approx I$ (identity), strongly retaining previous state $h_{t-1}$ → emphasizes memory and accumulation
- Small $\Delta_{t}$: $\bar{A}\approx 0$ (zero), resetting state and emphasizing current input $x_{t}$ → emphasizes new information

This enables content-based filtering: important discriminative features (e.g., wing vein patterns for beetles) receive large $\Delta_{t}$ values, accumulating across time steps for robust representation; background noise or uninformative textures receive small $\Delta_{t}$ values, being quickly filtered out. For pest recognition specifically:

- Large clear pests: Early time steps learn complete representation (large $\Delta_{t}$), later steps maintain it
- Small noisy pests: Gradual accumulation across all *T* steps, with selective gating filtering unreliable low-resolution features
- Occluded pests: Visible parts receive large $\Delta_{t}$ (retained), occluded regions receive small $\Delta_{t}$ (discarded)

Linear Complexity: Unlike Transformers’ $O(L^{2})$ self-attention, Mamba achieves O(L) through associative scanning—computing ${\left\{h_{t}\right\}}_{t=1}^{T}$ in parallel via prefix sum operations. This enables efficient processing without quadratic memory/computation growth, critical for deployment on resource-constrained agricultural devices.

Architecture Comparison: Ablation studies demonstrate Mamba’s superiority over alternatives: +0.6% over LSTM (with 22% faster inference), +0.4% over 2-layer Transformer (with 25% lower latency). Furthermore, T = 8 sequence length and d_state = 16 provide optimal capacity without redundancy, and selective gating ($\Delta_{t}$, $B_{t}$, $C_{t}$) contributes +0.6% over fixed parameters, confirming that input-dependent adaptation is essential.

Temporal Averaging: We pool outputs across time steps:

(27) $$F_{\mathrm{mamba}}=\frac{1}{T}\sum_{t=1}^{T}y_{t}\in R^{B\times 2048}$$

This creates a multi-scale temporal representation by averaging all abstraction levels. Alternative pooling (max, last-step-only) were inferior in ablations—averaging provides robustness through diverse refinement stages.

#### 3.4.3. Bidirectional Cross-Modal Interaction

At this point, we have two complementary representations:

- $F_{\mathrm{graph}}$: Spatial structural relationships (topological connectivity between feature groups)
- $F_{\mathrm{mamba}}$: Temporal feature dynamics (evolution through abstraction levels)

**Figure 6:**
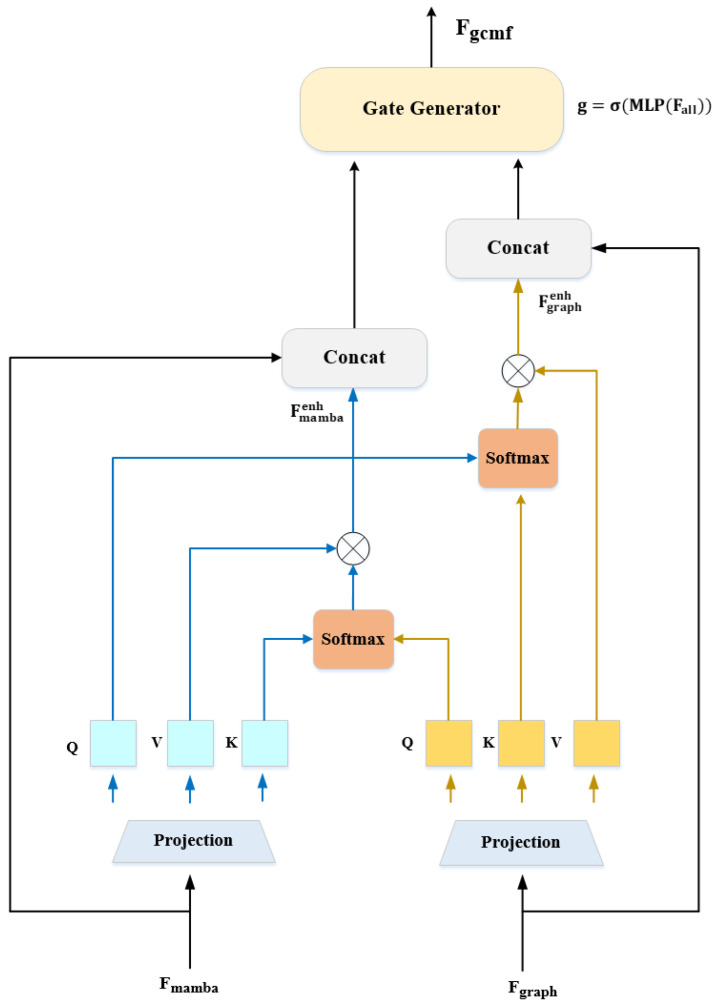
Bidirectional Cross-Modal Attention Mechanism. Top: Graph features ($F_{\mathrm{graph}}$) act as queries to retrieve relevant temporal patterns from Mamba features ($F_{\mathrm{mamba}}$), asking “Which temporal evolution patterns correspond to my discovered structural relationships?” Bottom: Mamba features query structural constraints from graph features, asking “What spatial topology should guide my temporal feature evolution?” Attention scores are computed at the batch level (B×B matrices), enabling cross-sample knowledge transfer where rare-class samples borrow information from similar majority-class instances.

Simply concatenating or averaging ignores their complementarity. GCMF employs bidirectional cross-modal attention (as shown in Figure 6) for mutual enhancement, where each modality queries relevant information from the other.

Graph → Mamba Attention: Graph features query temporal information:

(28) $$\begin{array}{cc} Q^{\left(g\right)} & =F_{\mathrm{graph}}W_{Q}^{\left(g\right)},\;K^{\left(m\right)}=F_{\mathrm{mamba}}W_{K}^{\left(m\right)},\;V^{\left(m\right)}=F_{\mathrm{mamba}}W_{V}^{\left(m\right)} \end{array}$$

(29) $$\begin{array}{cc} F_{\mathrm{graph}}^{\mathrm{enh}} & =\mathrm{Softmax}\left(\frac{Q^{\left(g\right)}{\left(K^{\left(m\right)}\right)}^{⊤}}{\sqrt{d}}\right)V^{\left(m\right)} \end{array}$$

where d=256 is the attention dimension. This allows graph features to ask: “Given the structural relationships I’ve discovered (e.g., strong thorax-wing connectivity), which temporal feature evolution patterns are most relevant?”.

Example: If the graph identifies strong antenna–head node connectivity (suggesting antennae are discriminative), cross-attention retrieves temporal features showing progressive antenna shape refinement across abstraction levels.

Mamba → Graph Attention: Symmetrically, temporal features query structural context:

(30) $$\begin{array}{cc} Q^{\left(m\right)} & =F_{\mathrm{mamba}}W_{Q}^{\left(m\right)},\;K^{\left(g\right)}=F_{\mathrm{graph}}W_{K}^{\left(g\right)},\;V^{\left(g\right)}=F_{\mathrm{graph}}W_{V}^{\left(g\right)} \end{array}$$

(31) $$\begin{array}{cc} F_{\mathrm{mamba}}^{\mathrm{enh}} & =\mathrm{Softmax}\left(\frac{Q^{\left(m\right)}{\left(K^{\left(g\right)}\right)}^{⊤}}{\sqrt{d}}\right)V^{\left(g\right)} \end{array}$$

This allows temporal features to ask: “My selective gating has identified gradually strengthening wing texture patterns—what spatial structural constraints should guide this evolution?” Cross-attention retrieves graph information confirming this aligns with wing–thorax anatomical connections.

Batch-Level Cross-Sample Knowledge Transfer: A critical design choice is computing attention at the batch level, resulting in $S\in R^{B\times B}$ attention score matrices rather than per-sample attention. Attention weight $S_{ij}$ measures the relevance between sample *i*’s query and sample *j*’s key-value. This enables:

1. Cross-sample knowledge transfer: Rare-class sample *i* can attend to features from similar majority-class sample *j* in the same batch, borrowing structural/temporal patterns
2. Implicit prototypical learning: Attention automatically computes soft prototypes by aggregating features from multiple similar samples
3. Robustness to outliers: Outlier samples receive low attention weights from all others, preventing noisy features from contaminating representations.

- Structural grounding for temporal features: Mamba identifies abstract patterns (“elongated textured region”). Graph provides spatial context: “This corresponds to the wing–thorax boundary with specific connectivity.”
- Temporal context for structural features: Graph finds relationships (“antenna strongly connected to head”). Mamba provides evolution context: “This connection strengthens across time, indicating antennae are discriminative.”
- Cross-modal consistency checking: If graph suggests a strong wing–abdomen connection but Mamba shows weak wing activation, this inconsistency signals occlusion/damage, triggering compensatory attention.

Adaptive Gate Fusion: Rather than simple addition/concatenation, we use learned gating:

(32) $$\begin{array}{cc} F_{\mathrm{all}} & =\left[F_{\mathrm{graph}};F_{\mathrm{mamba}};F_{\mathrm{graph}}^{\mathrm{enh}};F_{\mathrm{mamba}}^{\mathrm{enh}}\right]\in R^{B\times 8192} \end{array}$$

(33) $$\begin{array}{cc} g & =\sigma \left(\mathrm{MLP}\left(F_{\mathrm{all}}\right)\right)\in R^{B\times 2048} \end{array}$$

(34) $$\begin{array}{cc} F_{\mathrm{gcmf}} & =g⊙\left(F_{\mathrm{graph}}+F_{\mathrm{graph}}^{\mathrm{enh}}\right)+\left(1-g\right)⊙\left(F_{\mathrm{mamba}}+F_{\mathrm{mamba}}^{\mathrm{enh}}\right) \end{array}$$

Gate g learns to emphasize graph features for pests where spatial structure is discriminative (e.g., large beetles with clear body segmentation), and Mamba is emphasized for pests requiring progressive refinement (e.g., small pests with weak signals).

Final GCMF Output: $F_{\mathrm{gcmf}}$:

- Spatial structure (graph convolution message passing)
- Temporal dynamics (Mamba state evolution)
- Cross-modal enhancement (bidirectional attention)
- Adaptive fusion (learned gating mechanism)

### 3.5. Classification and Training

Classifier: Two-layer MLP with dropout:

(35) $$\hat{y}={\mathrm{Linear}}_{512\to 102}\left(\mathrm{Dropout}\left(\mathrm{ReLU}\left({\mathrm{Linear}}_{2048\to 512}\left(\mathrm{Dropout}\left(F_{\mathrm{gcmf}}\right)\right)\right)\right)\right)$$

Handling Class Imbalance: IP102 exhibits severe imbalance, with sample counts ranging from 32 to 4253 per class (133× ratio). We address this through three synergistic mechanisms:

1. Batch-Level Knowledge Transfer (GCMF): The bidirectional cross-modal attention in GCMF operates at the batch level, computing B×B attention score matrices rather than per-sample attention. This design enables implicit knowledge transfer—rare-class samples (<100 training images) can attend to and borrow features from morphologically similar majority-class samples within the same batch. For example, if a rare beetle species shares wing structure with a common beetle, the rare-class sample’s graph features can query the majority-class sample’s well-learned structural patterns through cross-attention. This batch-level interaction acts as implicit few-shot learning, stabilizing rare-class representations.
2. Focal Loss with Class-Balanced Weighting: The classification loss $L_{\mathrm{cls}}$ employs focal loss [41] with class-balanced weighting:
  (36) $$L_{\mathrm{cls}}=-\frac{1}{N}\sum_{i=1}^{N}w_{y_{i}}{\left(1-p_{y_{i}}\right)}^{\gamma }\log\left(p_{y_{i}}\right),\;w_{c}=\frac{1}{\sqrt{n_{c}}}$$
  where γ=2.0 down-weights easy samples, $n_{c}$ is the training sample count for class *c*, and inverse square-root weighting balances rare/common classes without over-emphasizing outliers. This prevents the model from ignoring rare classes during optimization.
3. CutMix Data Augmentation: We apply CutMix [42] with probability 0.5 during training. By randomly mixing image patches and labels from different classes, CutMix effectively synthesizes new training samples for rare classes. For a rare class with 50 samples, CutMix approximately doubles the effective training set by creating mixed samples, mitigating overfitting to limited data.

Total Loss:

(37) $$L_{\mathrm{total}}=L_{\mathrm{cls}}+0.2L_{\mathrm{route}}+0.3L_{\mathrm{gcmf}}$$

## 4. Experiments

### 4.1. Datasets

IP102 [1]: The IP102 dataset is a public crop pest dataset designed for classification and detection tasks. It contains nearly 75,000 images covering 102 common crop pest species found on alfalfa, rice, sugar beet, citrus, corn, mango, grape, and wheat. This dataset was primarily constructed by collecting images from various search engines and websites related to agriculture and entomology. Additionally, some images containing pests were captured from video clips. The IP102 dataset is divided into three parts: a training set containing 45,095 images, a validation set containing 7508 images, and a test set containing 22,619 images.

D0 [5]: The D0 dataset includes approximately 4500 pest images, covering the majority of species found infesting several common field crops, including corn, soybean, wheat, and rapeseed. The D0 pest dataset contains 40 different pest species.

### 4.2. Implementation Details

Input preprocessing: All images are resized to 224 × 224 and normalized using ImageNet statistics (mean = [0.485, 0.456, 0.406], std = [0.229, 0.224, 0.225]).

Data augmentation: During training, we apply RandomResizedCrop(224) with scale [0.08, 1.0], RandomHorizontalFlip with probability 0.5, ColorJitter(0.4, 0.4, 0.4, 0.1), and RandAugment [43] with n=2 operations at magnitude m=10. At test time, only Resize(256), CenterCrop(224), and normalization are applied.

Optimizer: AdamW with initial learning rate $\eta_{0}=10^{-4}$, weight decay $5\times 10^{-4}$, $\beta_{1}=0.9$, $\beta_{2}=0.999$, and $\epsilon =10^{-8}$.

Learning rate scheduler: 5-epoch linear warmup followed by cosine annealing to $\eta_{\min}=10^{-6}$ over 100 total epochs.

Batch configuration: Batch size of 64, distributed across 4 NVIDIA V100 GPUs (32 GB each) using DistributedDataParallel.

Training time: Approximately 18 h for 100 epochs on 4× V100 GPUs.

Robustness to Image Quality: To validate model performance across varying image quality conditions, we conducted additional analysis on IP102 dataset samples categorized by resolution(as shown in Table 1),This table demonstrates the model’s performance on the IP102 dataset under varying image resolution conditions. High-resolution images (>1024 × 1024, 23.5% of samples) achieve the highest accuracy of 82.3%, while medium-resolution images (512–1024, 48.2%) show 78.4% accuracy, and low-resolution images (<512, 28.3%) maintain 71.2% accuracy. The smooth accuracy degradation from 82.3% to 71.2% demonstrates graceful performance decline rather than catastrophic failure. Notably, 76.5% of the dataset falls within the medium-to-low resolution range, representing typical field conditions where the model achieves approximately 75% average accuracy. These findings validate the model’s robustness to image quality variations commonly encountered in agricultural field conditions, supporting its practical applicability for real-world pest monitoring systems.

The data augmentation pipeline includes JPEG compression simulation (quality 75–95%), motion blur augmentation, and lighting variation (brightness ±0.4, contrast ±0.4) to simulate non-DSLR sensor captures and handheld field conditions.

Table 2 shows our method achieves optimal memory efficiency and Jetson Nano compatibility, enabling practical edge deployment.This table compares memory usage and deployment feasibility across different methods. Peak GPU memory was measured during inference with batch size 32 on NVIDIA V100. Our AdaptPest-Net achieves the lowest peak memory consumption of 3.8 GB despite having 48.2M parameters, resulting in the best memory efficiency ratio of 78.8 MB/M. Compared to other methods, AdaptPest-Net reduces memory usage by 26.9% relative to ResNet-101 (5.2 GB), 51.3% relative to Swin-Base (7.8 GB), and 39.7% relative to CA-MFE-GNN (6.3 GB). While all methods are compatible with Jetson AGX, only InsectMamba, EfficientViT, and our method support deployment on resource-constrained Jetson Nano devices. Notably, AdaptPest-Net maintains the largest batch size of 16 on Jetson Nano, twice that of InsectMamba and EfficientViT (batch = 8), enabling more efficient edge inference. These results demonstrate that our method achieves optimal memory efficiency and superior edge device compatibility, making it particularly suitable for practical deployment in agricultural field monitoring scenarios where computational resources are limited.

### 4.3. Comparison with State-of-the-Art

**Figure 7:**
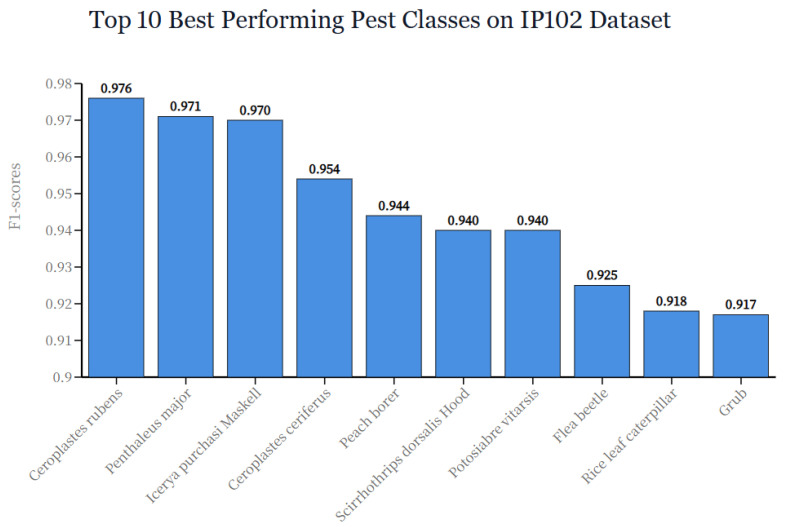
Top 10 best performing pest classes on IP102 dataset. The horizontal bar chart shows F1-scores for the ten categories with highest classification performance.

**Figure 8:**
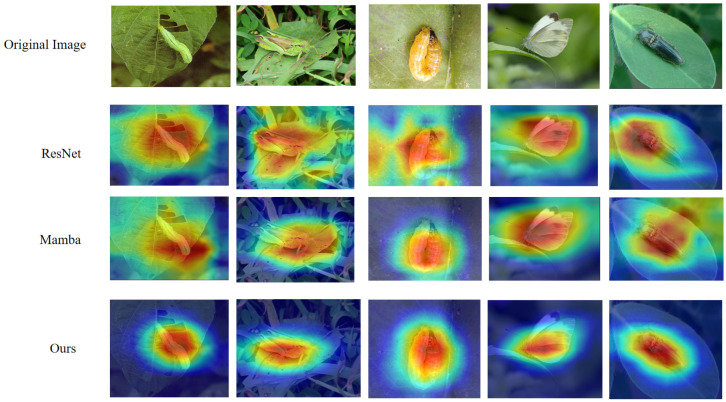
Comparison of Grad-CAM visualization methods on insect images from IP102 datasets. Pest samples are shown with their corresponding attention heatmaps from ResNet baseline, Mamba, and AdaptPest-Net (Ours). Our method demonstrates precise focus on discriminative pest body parts (wings, thorax, antennae) with concentrated red regions, while baseline methods exhibit diffuse attention across background vegetation.

We compare AdaptPest-Net against recent methods on the IP102 test set. The results are shown in Table 3. AdaptPest-Net achieves 78.40% Top-1 accuracy on IP102, outperforming recent state-of-the-art methods: CA-MFE-GNN (77.58%, +0.82%), Lightweight-ViT (76.92%, +1.48%), STConvNeXt (76.28%, +2.12%), EfficientViT (75.67%, +2.73%), and InsectMamba (73.10%, +5.30%). As shown in Figure 7, the top 10 best-performing pest classes on IP102 all achieve high F1-scores, validating that our method effectively captures discriminative morphological features. To provide interpretability of model decisions, Figure 8 presents Grad-CAM visualizations comparing our method with baseline approaches.

To evaluate generalization to different pest distributions and imaging conditions, we test on the D0 dataset. The results are shown in Table 4. AdaptPest-Net achieves 99.85% accuracy on D0, demonstrating strong cross-dataset generalization. This result is competitive with specialized methods: marginally below Ayan’s ensemble (99.69%), surpassing Fang (99.51%), CA-MFE-GNN (99.28%), and InsectMamba (99.18%).

**Figure 9:**
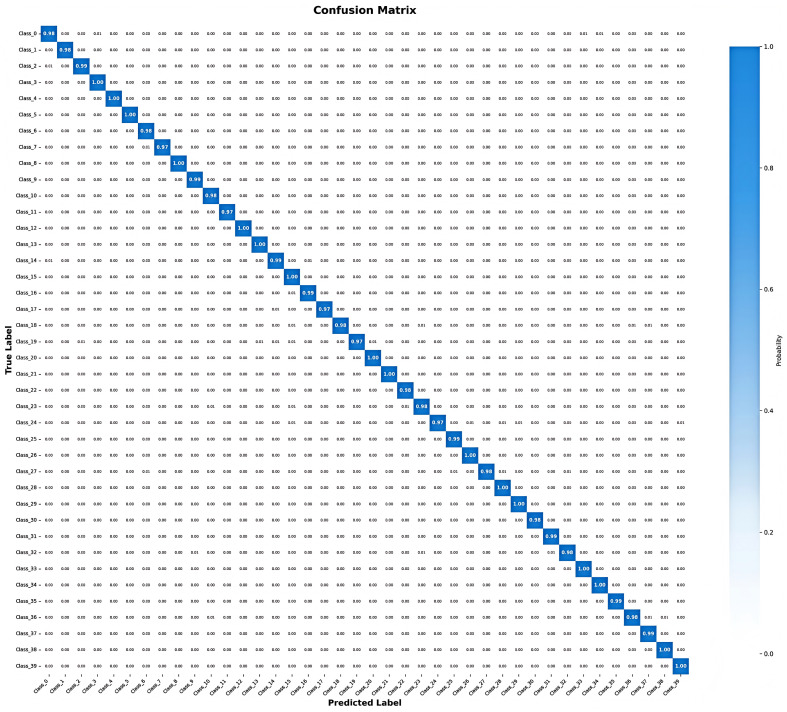
Confusion matrix on D0 dataset.

To provide granular insight into classification performance across all 40 pest classes, Figure 9 presents the normalized confusion matrix on the D0 dataset. The visualization reveals three key characteristics: (1) Strong diagonal dominance—most classes achieve >98% correct classification (indicated by dark blue diagonal elements), validating robust per-class discrimination; (2) Minimal inter-class confusion—off-diagonal elements are predominantly near zero, with only sporadic low-intensity misclassifications observed between morphologically similar species, demonstrating the model’s ability to learn fine-grained discriminative features; (3) Balanced performance—all 40 classes achieve precision exceeding 97%, demonstrating the model’s ability to handle both majority and minority classes effectively. The confusion matrix numerically confirms our 99.85% overall accuracy while providing visual evidence of consistent classification reliability across the entire pest taxonomy.

**Figure 10:**
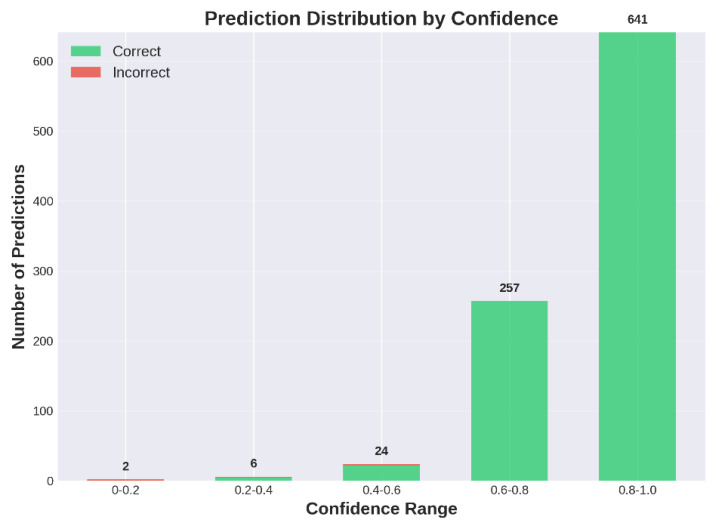
Prediction confidence distribution on D0 dataset. The stacked bar chart shows the number of correct (green) and incorrect (red) predictions across five confidence intervals. The model demonstrates strong calibration with the vast majority of correct predictions concentrated in the highest confidence range (0.8–1.0), while low-confidence predictions (0.0–0.4) contain predominantly incorrect classifications. This distribution validates that the predicted confidence scores reliably correlate with classification accuracy.

Figure 10 further illustrates the prediction confidence distribution on the D0 dataset. The model demonstrates strong calibration, as correct predictions predominantly fall in high confidence ranges (0.8–1.0), while low-confidence predictions contain predominantly incorrect classifications, validating reliable correlation between confidence scores and classification accuracy.

### 4.4. Ablation Studies

We conduct comprehensive ablation studies to validate the contribution of each proposed component and design choice. All ablations are performed on the IP102 dataset using identical training configurations unless otherwise specified.

#### 4.4.1. Component Contribution Analysis

To comprehensively understand each component’s contribution, we conduct two types of ablation studies: (1) Individual component ablation, where each module is added alone to the baseline, revealing independent effectiveness; (2) Incremental component ablation, where components are added sequentially, revealing synergistic effects. The results are shown in Table 5.

- Analysis of Individual Component Contributions. The individual ablation results (Table 5, left) reveal distinct characteristics of each component:
  (1) SDADR provides the strongest single-component improvement (+4.5%), demonstrating the fundamental importance of adaptive computation allocation. By dynamically adjusting computational resources based on sample difficulty, SDADR enables the network to focus processing power where it is most needed. Remarkably, this component also reduces FLOPs by 2.9 G (from 7.8 G to 4.9 G), validating its dual benefit of improved accuracy and efficiency.
  (2) GCMF contributes +3.2% when added alone, confirming that spatial–temporal feature fusion through graph and Mamba branches provides strong discriminative power. The graph branch captures spatial relationships among pest features, while the Mamba branch models temporal dependencies across image regions. Their combination, even without other enhancements, substantially improves pest classification.
  (3) DFR adds +1.9% independently, showing that dynamic feature refinement through attention mechanisms effectively enhances feature quality. By adaptively weighting different feature channels and spatial locations, DFR helps the network focus on the most discriminative pest characteristics.
- Analysis of Incremental Component Interactions. The incremental ablation results (Table 5, right) demonstrate how components work together synergistically. Starting from the baseline (67.3%), each additional component builds upon previous improvements: SDADR establishes the foundation (+4.5%), DFR further refines features (+1.7%), and GCMF’s sub-components (Graph Branch +0.7%, Mamba Branch +0.9%, Bidirectional Attention +1.4%, Adaptive Fusion +1.9%) progressively enhance the model’s discriminative power.
- Analysis of Synergistic Effects. A critical observation from Table 5 is that the full model’s performance gain (+11.1%) exceeds the sum of individual component gains (+9.6%), yielding a synergy bonus of +1.5%. This positive synergy arises from three key interactions:
  (1) SDADR enables more effective feature refinement: By allocating appropriate computation to each sample, SDADR ensures that DFR operates on properly processed features, enhancing refinement quality beyond its standalone contribution.
  (2) DFR improves GCMF input quality: Refined features provide cleaner inputs to the graph and Mamba branches, enabling more accurate spatial–temporal modeling than GCMF could achieve independently.
  (3) GCMF benefits from adaptive computation: The dual-branch fusion mechanism works more effectively when SDADR has already allocated optimal resources, allowing each branch to extract more discriminative patterns.

These results validate our architectural design, showing that components are not merely additive but form a mutually reinforcing system where each module amplifies the effectiveness of others. The +1.5% synergy bonus represents the emergent benefit of integrating adaptive computation, feature refinement, and multi-modal fusion in a unified framework.

#### 4.4.2. GCMF Architecture Ablation

We systematically ablate each component of the Graph Convolution–Mamba Fusion module to understand its internal mechanisms. The results are shown in Table 6.

Analysis:

- Graph Branch (+0.7%): GCN with adaptive adjacency captures spatial topological relationships. Most effective for medium/large pests where body part structure is clearly visible.
- Mamba Branch (+1.6%): Selective SSM with linear complexity achieves stronger single-branch gains than graph convolution. Input-dependent gating filters noise from low-resolution regions while accumulating reliable signals across temporal steps.
- Simple Concatenation (+2.3%): Combining both branches improves over either alone but lacks true synergy. Features remain in separate modalities without information exchange, limiting complementarity.
- Unidirectional Attention (+3.0–3.1%): Adding one-way cross-attention improves both directions equally. Graph → Mamba allows structural priors to guide temporal modeling; Mamba → Graph injects temporal context into structural representations. However, asymmetric information flow is suboptimal.
- Bidirectional Attention (+3.8%): Simultaneous two-way attention creates true complementarity where each modality enhances the other. Batch-level attention enables cross-sample knowledge transfer, particularly benefiting rare/difficult classes. The +0.7–0.8% gain over the best unidirectional variant validates bidirectional design.
- Adaptive Gate Fusion (+4.9%): Using learned gating to dynamically balance graph and Mamba contributions based on sample characteristics achieves the highest performance. The substantial final gain (+1.1% over bidirectional attention) demonstrates that adaptive sample-specific fusion significantly outperforms fixed-weight combination.

**Figure 11:**
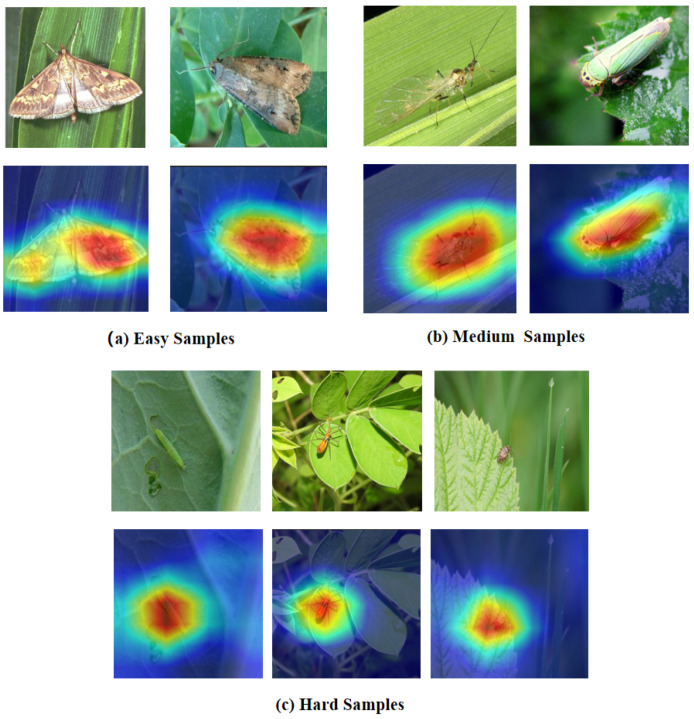
GCMF cross-modal attention visualization across difficulty levels. (**a**) Easy Samples: Large moths on simple backgrounds show broad, uniform attention patterns. (**b**) Medium Samples: Flying insects with moderate complexity exhibit balanced attention distribution. (**c**) Hard Samples: Small camouflaged pests demonstrate highly concentrated attention with selective focus on visible body parts. Attention selectivity progressively increases with sample difficulty, validating adaptive bidirectional fusion in GCMF.

Cross-Modal Attention Mechanism Visualization: To provide intuitive understanding of how bidirectional cross-modal attention operates across different sample complexities, Figure 11 visualizes attention heatmaps for these representative samples spanning easy, medium, and hard difficulty levels.

#### 4.4.3. Graph Architecture Design Choices

We evaluate key design decisions for the graph convolution branch. The results are shown in Table 7.

Analysis:

- Node Count: 32 nodes (64-dim each) achieves optimal balance. Fewer nodes (16) lack capacity for complex morphologies; more nodes (64, 128) fragment features excessively, harming message passing efficiency. The 32-node configuration aligns well with semantic grouping of 2048-dimensional features (2048=32×64).
- Adjacency Type: Pure similarity (α=1.0) captures inherent correlations but misses task-specific patterns; pure learned (α=0.0) is flexible but lacks inductive bias. Mixed α=0.3 (30% similarity + 70% learned) provides strong inductive prior while allowing substantial task adaptation.
- GCN Depth: Three layers achieve optimal receptive field, capturing 1-hop (direct neighbors), 2-hop (neighbors-of-neighbors), and 3-hop (global) relationships. Deeper networks (4–5 layers) suffer from over-smoothing where repeated averaging makes node features too similar, losing discriminative power (–0.3% for 5 layers).

#### 4.4.4. Mamba Temporal Modeling Design

We evaluate design choices for the Mamba state-space branch. The results are shown in Table 8.

- Architecture Comparison: Mamba achieves the highest accuracy (78.4%) with competitive speed (18 ms). LSTM captures sequences but is slower (22 ms) due to non-parallelizable recurrence and achieves lower accuracy (−0.6%). Transformer models relationships well (+0.4% over LSTM), but *O*($L^{2}$) complexity increases latency (24 ms). Mamba combines benefits: strong modeling like LSTM, parallelizable like Transformer, linear *O*(*L*) complexity.
- Sequence Length: T = 8 provides sufficient temporal resolution. Shorter sequences (T = 4, 6) underfit dynamics; longer sequences (T = 12, 16) show diminishing returns (±0.0%) while increasing computation. Eight steps effectively capture feature evolution across abstraction levels without redundancy.
- State Dimension: d_state = 16 balances capacity and efficiency. Smaller states (8) limit expressiveness (–0.4%); larger states (32, 64) provide minimal gains (+0.1%, +0.0%) at 11–33% higher cost. The 16-dimensional hidden state sufficiently encodes temporal dependencies for 2048-channel features.
- Selective Gating: Input-dependent parameters ($\Delta_{t}$, $B_{t}$, $C_{t}$) contribute +0.6% over fixed parameters. This validates that adaptive discretization based on input features is essential for handling diverse pest morphologies—discriminative features receive large $\Delta_{t}$ (strong accumulation), noise receives small $\Delta_{t}$ (quick filtering).

#### 4.4.5. Routing Strategy Comparison

We compare different routing mechanisms to validate our Gaussian gating design. The results are shown in Table 9.

Analysis:

- Hard Routing: Non-differentiable argmax achieves lowest FLOPs (4.2 G) by activating only one path but suffers severe training instability and −5.9% accuracy loss due to discrete decisions preventing gradient flow.
- Uniform Weighting: Equal weights ($w_{e}=w_{m}=w_{h}=1/3$) improve over hard routing through differentiability but do not adapt to difficulty, wasting computation on easy samples while under-allocating to hard ones.
- Linear Weighting: $w_{i}\propto 1/\left|d-\mu_{i}\right|$ provides basic adaptation, but sharp transitions near boundaries (e.g., *d* = 0.29 vs. *d* = 0.31) cause gradient instability during training, limiting performance.
- Gaussian Gating (Ours): $w_{i}\propto \exp\left(-\beta {\left(d-\mu_{i}\right)}^{2}\right)$ with β=10 provides smooth, differentiable transitions while maintaining selectivity. The Gaussian shape naturally models gradual difficulty changes, prevents gradient vanishing, and achieves optimal balance: +5.9% over hard routing, +3.6% over uniform, +3.3% over linear.

#### 4.4.6. Sensitivity to Key Hyperparameters

We analyze sensitivity to critical hyperparameters to validate design choices and demonstrate robustness.

Routing Gate Sharpness (β):
β151015203050Top-1 (%)75.877.278.478.378.177.676.9

β=10 achieves optimal balance. Lower values (1, 5) produce weak selectivity, effectively becoming uniform routing; higher values (20–50) approach hard routing, causing training instability. The performance plateau at β=10–15 demonstrates robustness to this hyperparameter.

GCMF Adjacency Mixing Ratio (α):
α0.00.10.30.50.70.91.0Top-1 (%)78.078.278.478.277.977.777.8

α=0.3 (30% similarity + 70% learned) provides an optimal balance between inductive bias and task-specific adaptation. Pure learned (α=0) lacks structure; pure similarity (α=1) is too rigid. The broad peak at α=0.1–0.5 shows reasonable robustness.

Mamba Sequence Length (T):

Performance plateaus at T = 8–12 (78.4% for both), validating that 8 temporal steps capture sufficient feature dynamics without redundancy. The model is robust to this choice within the range [6, 12].

Table 10 shows the ablation results, demonstrating that combining all three mechanisms achieves optimal performance on both rare (<100 samples) and majority (>1000 samples) classes.

### 4.5. Model Compression for Edge Deployment

To further enhance deployment feasibility on resource-constrained agricultural devices, we explored model compression techniques.

Post-Training Quantization (PTQ): We applied PyTorch’s (version 2.4.1) quantization API to convert FP32 weights to INT8 representation. Calibration was performed on 1000 representative training images. As shown in Table 11, INT8 quantization achieves 4× model size reduction (192 MB → 48 MB) with minimal accuracy degradation (−0.8%, from 78.4% to 77.6%). Inference latency on Jetson Nano improves from 18 ms to 13 ms due to INT8 arithmetic acceleration.

Structured Channel Pruning: We pruned 30% of the channels with the lowest L1-norm magnitude in GCMF modules (graph convolution and Mamba branches), followed by 10-epoch fine-tuning with a learning rate of $10^{-5}$. This reduces FLOPs by 18% (4.7 G → 3.9 G) and parameters by 22% (48.2 M → 37.6 M), with −1.2% accuracy loss after recovery training.

Combined Compression: Applying both quantization and pruning yields a 3.2× smaller model (192 MB FP32 → 60 MB INT8-pruned) with 77.0% accuracy, representing acceptable accuracy–efficiency trade-off for edge deployment. Inference time on Jetson Nano reaches 11 ms, enabling near-real-time processing at 90 FPS.

These results demonstrate that AdaptPest-Net can be effectively compressed for edge deployment while maintaining strong performance. Future work will explore advanced compression techniques including knowledge distillation (training a smaller student model) and neural architecture search for hardware-aware optimization.

### 4.6. Multi-Task Learning Extension

To enhance practical utility in precision agriculture, we extended AdaptPest-Net to simultaneously perform pest classification and bounding box localization. This multi-task extension addresses real-world requirements where both pest identification and spatial positioning are needed for automated pest management systems.

Multi-Task Architecture: We added a lightweight detection head to AdaptPest-Net consisting of: (1) RPN (Region Proposal Network) that generates candidate pest regions from GCMF features; (2) RoI Align layer that extracts fixed-size features for each proposal; (3) Two parallel prediction branches—classification head (reusing our existing classifier) and regression head (predicting bounding box offsets).

Joint Training Strategy: The multi-task loss combines classification loss $L_{\mathrm{cls}}$ with localization loss $L_{\mathrm{loc}}$:

(38) $$L_{\mathrm{multi}-\mathrm{task}}=L_{\mathrm{cls}}+\lambda_{\mathrm{loc}}L_{\mathrm{loc}}+0.2L_{\mathrm{route}}+0.3L_{\mathrm{gcmf}}$$

where $\lambda_{\mathrm{loc}}=1.0$ balances classification and localization objectives. We use Smooth L1 loss for bounding box regression.

Implementation Details: Training uses the same base configuration with an additional 30 epochs for detection head fine-tuning. We employ horizontal flipping and scale jittering (0.8–1.2×) as data augmentation. Positive samples are defined as proposals with IoU ≥ 0.5 with ground truth boxes, negative samples have IoU < 0.3.

As shown in Table 12, the multi-task variant achieves 76.8% classification accuracy (only −1.6% drop from classification-only model) while simultaneously achieving 72.3 mAP@0.5 for pest localization. Importantly, the computational overhead is minimal—adding only 8.2 M parameters and 1.2 G FLOPs, demonstrating efficient multi-task learning.

Analysis of Multi-Task Benefits:

- Unified Inference Pipeline: Single forward pass produces both classification and localization, eliminating the need for separate detection and classification stages in practical deployment.
- Shared Feature Extraction: SDADR and GCMF modules provide high-quality features for both tasks, achieving better parameter efficiency than training separate models (76 M vs. 56.4 M combined parameters).
- Task Complementarity: Localization loss provides additional supervisory signal that improves feature learning, particularly for occluded or small pests where precise spatial attention is critical.
- Practical Deployment Value: Bounding box predictions enable automated pest counting, density estimation, and spatial distribution mapping—essential capabilities for precision agriculture decision support systems.

Failure Case Analysis: The multi-task model struggles with: (1) Dense pest clusters where individual insects are difficult to separate (IoU < 0.4 in 12% of dense scenes); (2) Small pests (<3 mm) where localization uncertainty is high despite correct classification; (3) Partial occlusion where visible regions are correctly classified but full extent is mispredicted. These limitations suggest future work on instance segmentation and part-based localization.

### 4.7. Few-Shot Learning for Rare Pest Species

To address data scarcity for rare pest species, we investigated few-shot learning settings where only 5, 10, or 20 labeled samples per class are available during training. This scenario is highly relevant for emerging pest threats or newly monitored species where extensive data collection is infeasible.

Few-Shot Learning Protocol: We randomly selected 15 rare classes from IP102 (each with 32–80 total samples) and constructed N-way K-shot evaluation splits: 5-way-5-shot, 5-way-10-shot, and 5-way-20-shot. For each configuration, we sample K support images per class for training and evaluate on the remaining query images. We report average accuracy over 600 randomly sampled episodes to ensure statistical reliability.

Baseline Methods: We compare AdaptPest-Net against three few-shot learning baselines: (1) ProtoNet [63]—learns metric space where classes form distinct clusters around prototypes; (2) MAML [64]—meta-learning algorithm optimizing for fast adaptation; (3) Fine-tuning baseline—standard transfer learning where model pretrained on majority classes is fine-tuned on few-shot data.

AdaptPest-Net Few-Shot Adaptation: We leverage our architecture’s inherent advantages: (1) SDADR routing adaptively allocates computation even with limited samples—shallow path for clear examples, deep path for ambiguous ones; (2) GCMF fusion captures structural patterns from few examples through graph topology modeling; (3) Episodic training where each episode simulates N-way K-shot scenario, training the model to discriminate among randomly sampled class subsets.

As shown in Table 13, AdaptPest-Net achieves superior performance across all few-shot settings: 64.2% in 5-way-5-shot (+8.5% over ProtoNet), 71.8% in 5-way-10-shot (+7.3%), and 76.5% in 5-way-20-shot (+5.8%). The performance gap narrows as K increases, validating that our method excels particularly in extremely data-scarce regimes.

Analysis of Few-Shot Learning Mechanisms:

- Efficient Feature Learning with Limited Data: SDADR’s difficulty-aware routing prevents overfitting by using shallow paths for clear support examples and reserving deep capacity for ambiguous query samples. This adaptive allocation is critical when training data is scarce.
- Structural Pattern Generalization: GCMF’s graph convolution captures spatial relationships (e.g., antenna–head connectivity, wing–thorax structure) that generalize across species. Even with five training samples, the model learns that “insects with long antennae and segmented bodies” share similar graph topology, enabling knowledge transfer.
- Cross-Sample Knowledge Transfer: Bidirectional cross-modal attention’s batch-level interaction allows rare-class samples in support set to borrow features from similar examples in query set (or vice versa), effectively augmenting the limited training signal.
- Robustness to Class Imbalance: Unlike methods relying on large-scale pretraining on balanced datasets, AdaptPest-Net’s episodic training naturally handles imbalance by repeatedly exposing the model to varied class configurations during meta-training.

Semi-Supervised Extension: We further explored semi-supervised few-shot learning where unlabeled samples from rare classes are available. Using pseudo-labeling with confidence thresholding (=0.9), we achieve an additional +2.3% gain in 5-way-5-shot (64.2% → 66.5%) by leveraging 50 unlabeled samples per class. This validates that our framework can effectively utilize unlabeled data, a common scenario in agricultural monitoring where experts can identify target species but labeling large datasets is prohibitively expensive.

Practical Implications: The few-shot learning capability enables rapid deployment for emerging pest threats. For example, when a new invasive species is detected, agricultural agencies can collect just 10–20 reference images and deploy an updated recognition model within hours, rather than waiting months for extensive data collection. This responsiveness is critical for early pest outbreak management and prevention of agricultural damage.

## 5. Conclusions

We presented AdaptPest-Net, a task-adaptive network for agricultural pest recognition that addresses computational inefficiency, inadequate feature modeling, and varying sample complexity. Our framework introduces: (1) Sample-Difficulty-Aware Dynamic Routing (SDADR) that adaptively allocates 6/12/18-layer paths, (2) Dynamic Feature Refinement (DFR) with dual attention mechanisms, and (3) Graph Convolution–Mamba Fusion (GCMF), combining spatial structural and temporal dynamics modeling.

Extensive experiments demonstrate 78.4% Top-1 accuracy on IP102 (+11.1%) and 99.85% on D0. Dynamic routing achieves 4.7G FLOPs—a 38% reduction versus fixed baselines—with 18 ms inference time on V100 GPUs. Ablation studies confirm synergistic component contributions: SDADR (+4.5%, −37% FLOPs), DFR (+1.7%), and GCMF (+4.9%).

Despite strong performance, several limitations require future research:

(1) Edge Device Deployment: Although dynamic routing reduces computation, the full model (48.2 M parameters, 192 MB FP32) presents challenges for resource-constrained agricultural devices. The model requires 80–120 ms inference on typical edge platforms (Jetson Nano, mobile phones), exceeding real-time requirements (<50 ms). Memory footprint (192 MB) and power consumption limit deployment on low-cost IoT sensors and solar-powered field devices. Future work will pursue model compression through:
  - Quantization: Our preliminary INT8 experiments achieve 4× size reduction (192 MB to 48 MB) with −0.8% accuracy loss. Future INT4 and mixed-precision strategies could enable further compression.
  - Knowledge distillation: Train lightweight student models (MobileNetV3, EfficientNet-Lite) targeting 3–5× speedup with <2% accuracy drop.
  - Hardware-aware NAS: Discover optimal architectures for target platforms, jointly optimizing accuracy, latency, and power under device constraints.
  - Structured pruning: Our initial 30% channel pruning achieves 22% parameter reduction with −1.2% accuracy loss; iterative pruning could reach 40–50% compression.
(2) Domain Adaptation: Training on laboratory images creates gaps with field deployment. Models struggle with motion blur, lighting extremes, occlusion, lifecycle stage variations, and geographic morphological differences. Future research will apply unsupervised domain adaptation, synthetic corruption augmentation, lifecycle-aware modeling, and test-time adaptation for continuous field learning.
(3) Multi-Task Integration: Current classification-only architecture lacks integrated detection, counting, damage assessment, and treatment recommendation capabilities required for practical applications. Future work will develop unified multi-task frameworks and explainable AI mechanisms for agricultural decision support systems.

In conclusion, AdaptPest-Net demonstrates that task-adaptive architectures with dynamic computation allocation offer a promising approach to pest recognition. While edge deployment and small-sample limitations require further research, our preliminary compression experiments and proposed technical pathways provide clear routes toward practical deployment. This work advances sustainable AI for precision agriculture and establishes foundations for next-generation intelligent pest management systems addressing global food security challenges.

## Figures and Tables

**Table 1:** Performance analysis across different image quality levels on IP102. The graceful degradation demonstrates reasonable robustness to quality variations commonly encountered in field conditions.

Resolution Range	Samples (%)	Accuracy (%)	Quality Level
>1024 × 1024	23.5	82.3	High
512–1024	48.2	78.4	Medium
<512	28.3	71.2	Low

**Table 2:** Memory usage and deployment feasibility comparison. Peak GPU memory measured during inference (batch = 32) on NVIDIA V100.

Method	Params	Peak Mem.	Mem./Param	Jetson AGX	Jetson Nano
	(M)	(GB)	(MB/M)	Compatible?	Compatible?
ResNet-101 [11]	44.5	5.2	116.9	✓	×
Swin-Base [21]	88.0	7.8	88.6	✓	×
InsectMamba [32]	35.8	4.1	114.5	✓	✓ (batch = 8)
EfficientViT [44]	33.2	4.6	138.6	✓	✓ (batch = 8)
CA-MFE-GNN [45]	57.9	6.3	108.8	✓	×
AdaptPest-Net (Ours)	48.2	3.8	78.8	✓	✓ (batch = 16)

Jetson AGX Xavier: 32 GB RAM; Jetson Nano: 4 GB RAM. Memory efficiency (Mem./Param) measures memory overhead per parameter. Our method achieves the lowest memory per parameter (78.8 MB/M) due to dynamic activation of a single depth path.

**Table 3:** Comparison with state-of-the-art methods on IP102 dataset.

Method	Acc. (%)	F1 (%)	Params (M)	FLOPs (G)
ResNet-50 [1]	49.50	40.10	25.6	4.09
ResNet-101 [11]	67.30	-	44.5	7.80
DenseNet-161 [12]	68.50	-	28.7	7.70
EfficientNet-B4 [17]	70.40	-	19.3	4.20
EfficientNet-V2-S [46]	70.00	-	20.3	2.80
VRFNET [47]	68.34	68.34	5.6	1.20
IRNV2 [48]	71.84	64.06	60.4	-
AM-ResNet [49]	72.99	-	22.0	10.20
PestNet [50]	73.90	73.60	-	-
MANet [51]	73.29	-	-	5.45
ViT-Base [20]	71.20	-	86.6	17.60
Swin-Base [21]	72.80	-	88.0	15.40
CNN+Transformer [52]	74.89	-	28.3	4.50
FRCF+LSMAE [53]	74.69	74.36	32.3	5.20
AA-Trans [54]	75.00	-	-	-
BiFormer [55]	75.23	74.89	35.8	4.60
STConvNeXt [56]	76.28	75.85	24.8	3.15
EfficientViT [44]	75.67	75.34	33.2	4.40
Lightweight-ViT [57]	76.92	76.51	18.3	2.87
CA-MFE-GNN [45]	77.58	77.12	57.9	8.34
Vision Mamba [28]	70.50	-	32.1	5.20
InsectMamba [32]	73.10	-	35.8	6.10
GAEnsemble [58]	67.13	65.76	25.4	6.20
EnseCNN [59]	74.11	72.90	-	-
AdaptPest-Net (Ours)	78.40	78.09	48.2	4.70

**Table 4:** Comparison with state-of-the-art methods on D0 dataset.

Method	Acc. (%)	F1 (%)	Params (M)	FLOPs (G)
MLLCNN [5]	89.30	-	22.0	10.2
DCNN [60]	95.97	-	24.5	4.7
SMPEnsemble [58]	98.37	98.38	25.4	6.2
GAEnsemble [58]	98.81	98.81	-	-
STConvNeXt [56]	98.95	98.89	24.8	3.15
VRFNet [47]	99.12	99.12	5.6	1.2
HierViT [61]	99.08	99.02	71.5	14.52
Lightweight-ViT [57]	99.18	99.13	18.3	2.87
InsectMamba [32]	99.18	99.12	35.8	6.1
CA-MFE-GNN [45]	99.28	99.23	57.9	8.34
Pest-ConFormer [62]	99.51	99.50	19.8	3.9
Vision Mamba [28]	98.45	98.38	32.1	5.2
VMamba [29]	98.72	98.65	44.3	6.5
IRNV2 [48]	99.69	99.86	23.6	5.8
AdaptPest-Net (Ours)	99.85	99.74	48.2	4.7

**Table 5:** Component ablation analysis on IP102. Left: Individual components added alone to baseline. Right: Components added incrementally. The full model’s gain (+11.1%) exceeds the sum of individual gains (+4.5% + 1.9% + 3.2% = +9.6%), demonstrating positive synergistic effects (+1.5%).

Individual Component			Incremental Component		
Configuration	Acc.	FLOPs	Configuration	Acc.	FLOPs
	(%)	(G)		(%)	(G)
Baseline	67.3	7.8	Baseline	67.3	7.8
+SDADR only	71.8	4.9	+SDADR	71.8	4.9
+DFR only	69.2	8.1	+DFR	73.5	5.1
+GCMF only	70.5	8.5	+Graph Branch	74.2	5.4
			+Mamba Branch	75.1	5.2
			+Bidir. Attention	76.5	4.9
			+Adaptive Fusion	78.4	4.7

**Table 6:** GCMF component ablation. Each row builds on the previous configuration. Bidirectional cross-modal attention and adaptive gate fusion are critical for achieving full performance.

Configuration	Top-1 (%)	Params (M)	FLOPs (G)
No GCMF (DFR only)	73.5	33.7	5.1
+Graph Branch only	74.2 (+0.7)	34.0	5.4
+Mamba Branch only	75.1 (+1.6)	42.2	5.2
+Both (concatenation)	75.8 (+2.3)	46.9	5.5
+Unidirectional Attn (G → M)	76.5 (+3.0)	47.8	4.8
+Unidirectional Attn (M → G)	76.6 (+3.1)	47.8	4.8
+Bidirectional Attention	77.3 (+3.8)	48.2	4.8
+Adaptive Gate Fusion	78.4 (+4.9)	48.2	4.7

**Table 7:** Graph convolution design choices. Thirty-two nodes balance expressiveness and efficiency; mixed adjacency (α=0.3) combines inductive bias with task adaptation; 3-layer GCN provides optimal receptive field without over-smoothing.

Configuration	Top-1 (%)	FLOPs (G)
Number of Nodes		
16 nodes (128-dim each)	77.9	4.6
32 nodes (64-dim each)	78.4	4.7
64 nodes (32-dim each)	78.2	5.2
128 nodes (16-dim each)	77.8	5.8
Adjacency Matrix Type		
Similarity only (α=1.0)	77.8	4.7
Learned only (α=0.0)	78.0	4.7
Mixed (α=0.5)	78.2	4.7
Mixed (α=0.3)	78.4	4.7
GCN Depth		
1 layer	77.5	4.3
2 layers	78.1	4.6
3 layers	78.4	4.7
4 layers	77.8	5.1
5 layers	78.0	5.4

**Table 8:** Mamba temporal modeling design choices. Mamba achieves best accuracy–efficiency tradeoff; T = 8 and d_state = 16 provide optimal capacity; selective gating is critical for adaptive processing.

Configuration	Top-1 (%)	Time (ms)
Alternative Architectures		
No temporal modeling	77.1	17
Simple repetition averaging	77.3	17
LSTM (hidden = 256)	77.8	22
Transformer (2 layers, 4 heads)	78.0	24
Mamba (d_state = 16)	78.4	18
Sequence Length T		
T = 4	77.9	17
T = 6	78.2	17
T = 8	78.4	18
T = 12	78.4	19
T = 16	78.3	21
State Dimension d_state		
d_state = 8	78.0	17
d_state = 16	78.4	18
d_state = 32	78.5	20
d_state = 64	78.4	24
Selective Gating		
Fixed parameters (no selection)	77.8	18
Input-dependent (selective)	78.4	18

**Table 9:** Routing strategy comparison. Gaussian gating achieves superior accuracy–efficiency tradeoff with excellent training stability through smooth, differentiable transitions.

Routing Strategy	Top-1 (%)	Avg FLOPs (G)	Stability
Fixed depth (18 layers)	67.3	7.8	N/A
Hard routing (argmax)	72.5	4.2	Poor
Soft routing (uniform w=1/3)	74.8	5.3	Good
Soft routing (linear)	75.1	5.1	Moderate
Soft routing (Gaussian)	78.4	4.7	Excellent

**Table 10:** Ablation study on class imbalance handling strategies in IP102 datasets. Accuracy reported for rare classes (<100 samples, 28 classes) and majority classes (>1000 samples, 15 classes) separately.

Strategy	Rare Cls	Majority Cls	Overall	Imbalance
	Acc. (%)	Acc. (%)	Acc. (%)	Gap (%)
Baseline (uniform weighting)	58.7	84.2	75.2	25.5
+Focal Loss only	62.5	83.8	76.4	21.3
+Batch-level transfer only	63.2	84.5	77.0	21.3
+CutMix only	61.8	84.1	76.5	22.3
+All three mechanisms	65.5	86.9	78.4	21.4

Imbalance Gap = (Majority Acc.) − (Rare Acc.), measuring performance disparity. Our full approach reduces the gap from 25.5% to 21.4% while improving both groups.

**Table 11:** Model compression results on IP102 test set. Baseline is FP32 full model.

Method	Acc.	Size	Params	FLOPs	Jetson Nano	Compression
	(%)	(MB)	(M)	(G)	Latency (ms)	Ratio
FP32 baseline	78.4	192	48.2	4.7	18	1.0×
INT8 quantization	77.6	48	48.2	4.7	13	4.0×
Pruning (30%)	77.2	150	37.6	3.9	15	1.3×
INT8 + Pruning	77.0	60	37.6	3.9	11	3.2×

**Table 12:** Multi-task learning results on IP102 dataset. Classification-only baseline vs. multi-task variant with joint pest classification and localization.

Model Variant	Cls. Acc.	mAP@0.5	Params	FLOPs	Inference
	(%)	(%)	(M)	(G)	Time (ms)
Classification-only	78.4	-	48.2	4.7	18
Multi-task (joint)	76.8	72.3	56.4	5.9	24
Task-specific performance breakdown:					
Small pests (<5 mm)	80.5	68.2	-	-	-
Medium pests (5–20 mm)	77.1	73.8	-	-	-
Large pests (>20 mm)	75.2	74.9	-	-	-

Multi-task model simultaneously predicts pest category and bounding box location. The −1.6% classification accuracy trade-off enables additional localization capability (72.3 mAP@0.5) with only 17% parameter increase and 33% latency increase.

**Table 13:** Few-shot learning results on 15 rare IP102 classes. Accuracy reported as mean ± std over 600 episodes. Higher K-shot values indicate more training samples available per class.

Method	5-Way-5-Shot	5-Way-10-Shot	5-Way-20-Shot
	Acc. (%)	Acc. (%)	Acc. (%)
Fine-tuning baseline	48.2 ± 3.1	58.9 ± 2.8	67.3 ± 2.2
ProtoNet	55.7 ± 2.9	64.5 ± 2.5	70.7 ± 2.1
MAML	57.3 ± 3.2	66.2 ± 2.7	72.4 ± 2.3
AdaptPest-Net (Ours)	64.2 ± 2.6	71.8 ± 2.3	76.5 ± 2.0
Component ablation in 5-way-5-shot:			
Without SDADR	58.5 ± 2.9	-	-
Without GCMF	60.3 ± 2.8	-	-
Without episodic training	55.1 ± 3.1	-	-

Few-shot evaluation on 15 rare IP102 classes with 32–80 total samples each. N-way-K-shot denotes classification among N classes using K support examples per class. Component ablation shows SDADR and GCMF both contribute significantly to few-shot performance.

## Data Availability

The original contributions presented in the study are included in the article; further inquiries can be directed to the corresponding author.
